# Columnar cell lesions of the canine mammary gland: pathological features and immunophenotypic analysis

**DOI:** 10.1186/1471-2407-10-61

**Published:** 2010-02-23

**Authors:** Enio Ferreira, Helenice Gobbi, Bruna S Saraiva, Geovanni D Cassali

**Affiliations:** 1 Department of Pathology, Biological Sciences Institute, Federal University of Minas Gerais, Av. Antônio Carlos, 6627, Belo Horizonte, Minas Gerais, 31270-901, Brazil; 2 Department of Anatomic Pathology, School of Medicine, Federal University of Minas Gerais, Av. Alfredo Balena, 190, Belo Horizonte, Minas Gerais, 30150-270, Brazil

## Abstract

**Background:**

It has been suggested that columnar cell lesions indicate an alteration of the human mammary gland involved in the development of breast cancer. They have not previously been described in canine mammary gland. The aim of this paper is describe the morphologic spectrum of columnar cell lesions in canine mammary gland specimens and their association with other breast lesions.

**Methods:**

A total of 126 lesions were subjected to a comprehensive morphological review based upon the human breast classification system for columnar cell lesions. The presence of preinvasive (epithelial hyperplasia and in situ carcinoma) and invasive lesions was determined and immunophenotypic analysis (estrogen receptor (ER), progesterone receptor (PgR), high molecular weight cytokeratin (34βE-12), E-cadherin, Ki-67, HER-2 and P53) was perfomed.

**Results:**

Columnar cell lesions were identified in 67 (53.1%) of the 126 canine mammary glands with intraepithelial alterations. They were observed in the terminal duct lobular units and characterized at dilated acini may be lined by several layers of columnar epithelial cells with elongated nuclei. Of the columnar cell lesions identified, 41 (61.2%) were without and 26 (38.8%) with atypia. Association with ductal hyperplasia was observed in 45/67 (67.1%). Sixty (89.5%) of the columnar cell lesions coexisted with neoplastic lesions (20 in situ carcinomas, 19 invasive carcinomas and 21 benign tumors). The columnar cells were ER, PgR and E-cadherin positive but negative for cytokeratin 34βE-12, HER-2 and P53. The proliferation rate as measured by Ki-67 appeared higher in the lesions analyzed than in normal TDLUs.

**Conclusions:**

Columnar cell lesions in canine mammary gland are pathologically and immunophenotypically similar to those in human breast. This may suggest that dogs are a suitable model for the comparative study of noninvasive breast lesions.

## Background

The development of human breast cancer is believed to be a complex multistep process originating in terminal duct lobular units (TDLUs) and progressing towards invasive cancer. Various precursor breast lesions have been implicated in cancer development: atypical ductal hyperplasia (ADH), atypical lobular hyperplasia (ALH), lobular carcinoma in situ (LCIS), ductal carcinoma in situ (DCIS) and more recently columnar cell lesions (CCLs) [1,2].

Columnar cell lesions (CCL) of the human breast comprise a group of conditions characterized by varying degrees of acinar dilation in the TDLUs, lined by several layers of columnar epithelial cells with uniform, ovoid nuclei oriented perpendicular to the basement membrane. The number of cellular layers enable CCLs to be divided into two broad categories: columnar cell change (CCC) [1-2 cell layers] or columnar cell hyperplasia (CCH) [>2 cell layers]. CCC and CCH with cytological atypia are further subclassified as flat epithelial atypia (FEA) [3-5].

Recent observational studies and emerging genetic evidence suggest that some CCLs, particularly those with low-grade/monomorphic-type cytological atypia, represent precursors to, or an early stage in the development of, low-grade ductal carcinoma in situ (DCIS) and invasive carcinoma [1,6].

The canine mammary gland bears significant pathological lesions similar to the human breast [7,8]. Breast lesions in dogs show cellular changes involved in the progression to invasive carcinoma. They are known as atypical hyperplasia and carcinoma in situ [9-11]. These descriptions may suggest that dogs are a promising model animal for comparative oncology. Therefore, a clearer account of the alterations in canine mammary cancer will help to better understand the key steps in the formation of human tumors.

In this paper we describe the presence of CCLs in canine mammary gland specimens, their association with other breast lesions and immunohistochemical findings in a series of specimens.

## Methods

### Specimen selection

Specimens from one hundred and twenty-six consecutive cases of canine mammary gland, with previous diagnosis of epithelial lesions, were selected from the archives of the Laboratory of Comparative Pathology of the Biological Science Institute of the Federal University of Minas Gerais. The mammary gland samples were obtained after clinical diagnosis of mammary tumor and surgical removal of the lesion. Hematoxylin and eosin-stained sections were reviewed to search for columnar cell and associated lesions. The ages of the animals at the time of surgery ranged from 3 to 16 years (mean 9.8 years ± 2.2).

One human pathologist (HG) and two veterinary pathologists (EF and GDC) individually reviewed and classified the CCL in terms of the Schnitt and Vincent-Salomon classification. Lesions were divided into two categories according to their distinguishing morphological features: columnar cell change (CCC) and columnar cell hyperplasia (CCH), including subclassifications of these according to the absence or presence of cytological atypia (Table 1) [4,5]. A consensus classification was achieved for each case by discussion and observation of each individual lesion on a multihead microscope. The canine mammary neoplasias and epithelial hyperplasias were classified according to veterinary nomenclature [12]. The association of CCL with malignant and benign lesions was analyzed using Fisher's exact test with significance at P < 0.05.

**Table 1:** Histologic features of the different categories of columnar cell lesions in canine mammary gland.

*Columnar Cell Change without atypia* *(CCC)*	*Columnar Cell Hyperplasia* *(CCH)*	*Flat Epithelial Atypia (FEA)*	*Flat Epithelial Atypia (FEA)*
		
		*CCC with atypia *	*CCH with atypia*
One to two columnar cell layers with uniform ovoid to elongated nuclei; nucleoli absent or inconspicuous.	Cellular stratification more than two columnar cell layers with uniform ovoid to elongated nuclei; nucleoli absent or inconspicuous.	One to two columnar cell layers with complex architectural patterns present; Mild to moderate cytologic atypia present (usually low-grade): round-to- ovoid, mildly pleomorphic and hyperchromatic nuclei, with inconspicuous nucleoli.	Cellular stratification more than two columnar cell layers with complex architectural patterns present; Mild to moderate cytologic atypia present (usually low-grade): round-to- ovoid, mildly pleomorphic and hyperchromatic nuclei, with inconspicuous nucleoli.

Classification categories are based on an expanded version of those describes by Schitt and Vincent- Salomon [5].

### Immunohistochemistry

Considering the small size of the lesions, the immunohistochemical analysis were performed only on cases with enough material. Paraffin blocks were selected from six cases containg columnar cell lesions. Consecutive 5 μm thick sections were obtained and mounted on silanated slides for immunohistochemical study. Sections were stained for rabbit polyclonal antibodies: HER-2 (c-erbB-2; Dako; dilution: 1:40), P53 (clone CM1; Covance; diluition: 1:80); and mouse monoclonal antibodies: E-cadherin (clone 4A2C7; Zymed; dilution: 1:100), ER-LH2 (clone CC4-5; Novocastra; dilution: 1:25), PgR (clone hPRa2; Neomarkers; dilution: 1:20), Ki-67 (clone Mib-1; Dako; dilution: 1:25), cytokeratins 1, 5, 10 and 14 (clone 34βE-12; Dako; dilution: 1:40). Heat-induced epitope retrieval (20 min) using Dako antigen retrieval solution, pH 6.0 (Dako), was previously performed in a water bath. The slides were then cooled to room temperature for 20 min in the antigen retrieval buffer. The sections were incubated at room temperature in 3% (vol/vol) H_2_O_2 _for 15 min, in primary antibodies for 16 h, in reagent contained anti-mouse and anti-rabbit secondary antibodies (Biotinylated Goat Anti-polyvalent, Laboratory Vision) for 15 min and streptavidin peroxidase (UltraVision Large Volume Detection System, HRP, Laboratory Vision) for 15 min. Between incubations, the slides were washed for 2 × 5 min in phosphate-buffered saline containing 1% (vol/vol) Tween 20. Immunoreactivity was visualized by incubating the slides for 10 min with diaminobenzidine (DAB Substrate System; Laboratory Vision). The slides were then counterstained with Harris hematoxylin. Positive and negative control slides were included in each batch. As a positive control we used human breast cancer tissue known to express of the antibodies. Negative controls were assessed using normal serum (Ultra V Block, Laboratory Vision) as the primary antibody.

Staining for ER, PgR, P53, CK34βE-12 and E-cadherin was evaluated semi-quantitatively and scored into five categories: negative (-), <5% of cells stained; positive (+), 5% to 25% of cells stained; positive (++), 25% to 50% of cells stained; positive (+++), 50% to 75% of cells stained; diffusely positive (++++), >75% of cells stained [13]. The proliferative index was calculated by counting the positive nuclei for Ki-67 staining in a total of 500 columnar cells from each lesion. HER-2 expression was defined as epithelial cell membrane staining and scored according to the American Society of Clinical Oncology, College of American Pathologists [14].

All procedures were performed under the guidelines and with the approval of the Ethics Committee in Animal Experimentation (CETEA/UFMG), protocol 192/2006.

## Results

**Figure 1:**
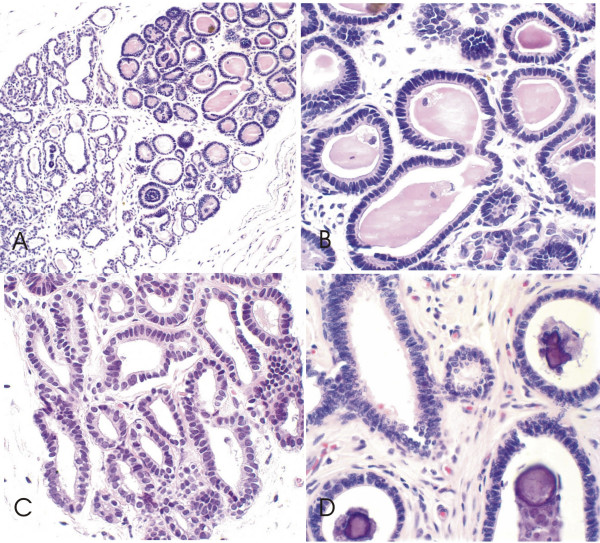
**Canine mammary biopsies with columnar cell change without atypia (CCC) H&E stain**. **1A **Low-power view, showing dilated mammary acini in a TDLU lined with a single layer of epithelial columnar cells with underlying myoepithelial cells, many of which contain intraluminal secretions and many hyperplastic foci, 200×; **1B, 1C e 1D **At higher magnification, the lining columnar cells with uniform ovoid to elongated nuclei and nucleoli absent. Many contain intraluminal secretions (**1B**) and show a small apical snout (**1C**) and intraluminal calcifications **(1D)**, 600×.

**Figure 2:**
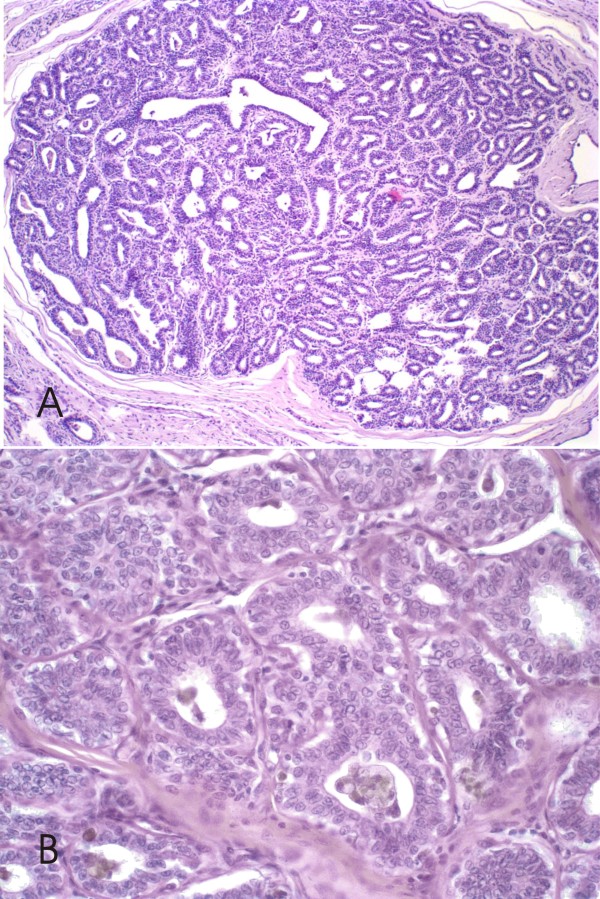
**Canine mammary biopsies with columnar cell hyperplasia without atypia (CCH) H&E stain**. **2A **Low-power view, terminal duct lobular unit with cellular stratification, more than two cell layers, 100×. **2B **Higher power view; columnar cells with uniform ovoid to elongated nuclei, observed hobnail cells with nucleoli absent or inconspicuous, 600×.

CCLs were identified in 67 (53.1%) canine mammary glands from the 126 specimens studied. CCL without atypia were identified in 41/67 (61.1%) canine mammary specimens. The CCC, 39 cases, were characterized by dilated acini lined with a single layer of columnar epithelial cells with elongated nuclei, a small amount of cytoplasm and apical cytoplasm frequently containing snouts and intraluminal secretions (Figure 1). Only two specimens the columnar lesions showed more than two cell layers and had prominent apical cytoplasmic snouts; these were classified as columnar cell hyperplasia without atypia (CCH) (Figure 2).

**Figure 3:**
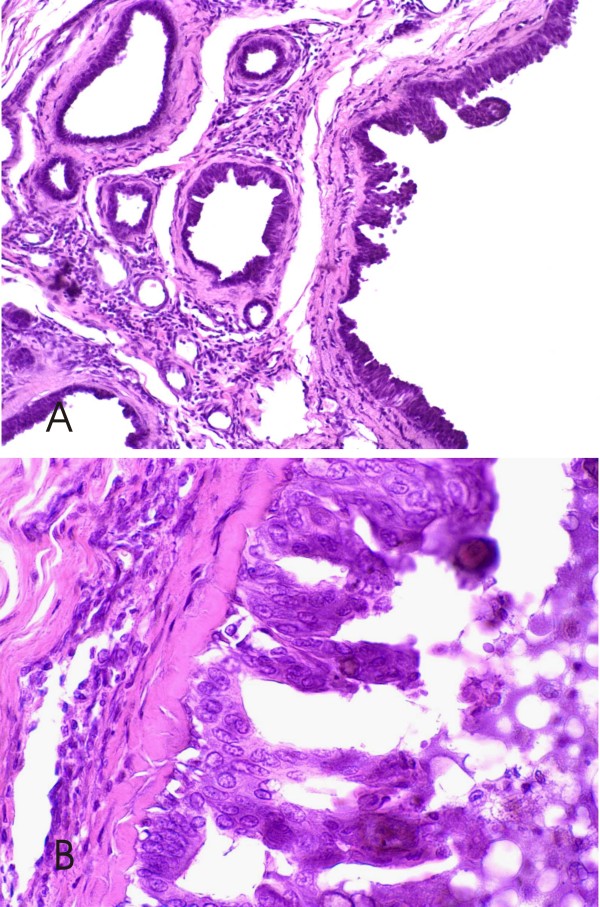
**Canine mammary biopsies with flat epithelial atypia (FEA) H&E stain**. **3A **Low-power view; terminal duct lobular unit shows larger acini with cellular atypia, prominent apical cytoplasmic snouts, to be lined by >2 cell layers. 200×; **3B **Higher power view; the columnar epithelial cells (> 2 layers) lining the acini with intraluminal secretions show cytological atypia, characterized by apical cytoplasmic snouts and enlarged, uniform nuclei; the nuclear/cytoplasmic ratio is increased. Nucleoli are evident in some of the nuclei, 600×.

FEA was found in 26/67 (38.8%) (24 cases of CCCs and 2 cases of CCHs) canine mammary lesions. In these cases the alterations were characterized by the presence of columnar epithelial cells with round to ovoid and/or hyperchromatic nuclei that were not perpendicularly oriented to the basement membrane, with a slight increase in the nuclear/cytoplasmic ratio (Figure 3). Focal micropapillae and tufting of cells were seen.

The intraluminal microcalcifications were detected in columnar cell changes (6 cases without atypia and 6 cases with atypia) and in columnar cell hyperplasia (only 1 case without atypia).

Associated lesions were represented only by ductal and lobular hyperplasias and epithelial neoplastic lesions. Sixty cases of the CCLs (89.5%) showed coexisting neoplastic lesions (20 ductal carcinomas *in situ*, 19 invasive carcinomas and 21 benign tumors). We found a higher prevalence of FEA with ductal carcinomas *in situ *(13 cases; 50%) than invasive carcinomas (5 cases; 19%) and benign tumors (3 cases; 11.5%). CCCs were detected in association with 18 benign tumors (46.1%), 7 ductal carcinomas *in situ *(17.9%) and 12 invasive carcinomas (30.7%) (P < 0.05). Two cases of CCH proved to be associated with invasive carcinoma (Table 2).

**Table 2:** Types of canine mammary columnar cell lesions and associated neoplastic alterations.

***Tumor types***^(**)^	***CCC(%)***	***CCH(%)***	***FEA(%)***	***Total(%)***
**invasive carcinomas**	**12 (30.7)**	-	**5 (19.2)**	**19 (28.4)**
*carcinoma in benign tumor*	*6 (15.3)*	*2 (100)*	*3 (11.5)*	
*solid carcinoma*	*2 (5.1)*	-	*2 (7.2)*	
*tubulopapillary carcinoma*	*4 (10.3)*	-	-	
**ductal carcinoma *in situ***	**7 (17.9)**	**-**	**13 (50.0)**^(*)^	**20 (29.8)**
**benign tumors**	**18 (46.1)**^(*)^		**3 (11.5)**	**21 (31.4)**
*simple adenoma*	*3 (7.6)*	-	**-**	
*duct papilloma*	*7 (17.9)*	-	*1 (3.8)*	
*benign mixed tumor*	*8 (20.6)*	-	*2 (7.7*)	
**without tumor**	**2 (5.1)**	**-**	**5 (19.2)**	**7 (10.4)**
**Total**	39 (100)	2 (100)	26 (100)	**67 (100)**

^(*)^Flat epithelial atypia presence is often associated with in situ carcinomas and columnar cell lesion without atypia are more frequent in benign tumors (P < 0,05).

^(**)^Misdorp,1999. *International Histological Classification of Tumors of Domestic Animals*.

CCC: columnar cell change without atypia; CCH: columnar cell hyperplasia without atypia; FEA: flat epithelial atypia

Ductal hyperplasia was associated with CCLs in 45/67 (67.1%) cases (23 without atypia and 22 with atypia) (Figure 1D). Two cases of CCHs was associated with ductal hyperplasia. Only one atypical lobular hyperplasia was detected. This lesion was associated with columnar cell hyperplasia without atypia and in situ carcinoma. FEA was associated with ductal hyperplasias with atypia in 12 out 17 cases and CCC was more frequently associated with hyperplasias without atypia: 17 out 26 cases (P < 0.05) (Table 3).

**Table 3:** Types of canine mammary columnar cell lesions and associated ductal hyperplasia in presence of different tumors types.

**Tumor types**^(**)^	**CCC(%)**		**CCH(%)**		**FEA(%)**		**Total(%)**
	
	***DH***	***ADH***	***DH***	***ADH***	***DH***	***ADH***	
**invasive carcinomas**	3 (11.5)	3 (11.5)	1 (50.0)	1 (50.0)	-	3 (17.6)	11 (24.4)
**ductal carcinoma in situ**	2 (7.7)	2 (7.7)	-	-	3 (17.6)	4 (23.5)	11 (24.4)
**benign tumors**	10 (38.5)	4 (15.4)	-	-	1 (5.9)	1 (5.9)	16 (35.6)
**without tumor**	2 (7.7)	-	-	-	1 (5.9)	4 (23.5)	7 (15.6)
**Total**	17 (65.4)^(*)^	9 (34.6)	1 (50.0)	1 (50.0)	5 (29.4)	12 (70.6)^(*)^	45 (100)

^(*)^Flat epithelial atypia is often associated with ductal hyperplasias with atypia (ADH) and columnar cell changes witout atypia are more frequent in hyperplasias without atypia (UDH) (19/30) (P < 0.05).

^(**)^Misdorp,1999. *International Histological Classification of Tumors of Domestic Animals*.

**Figure 4:**
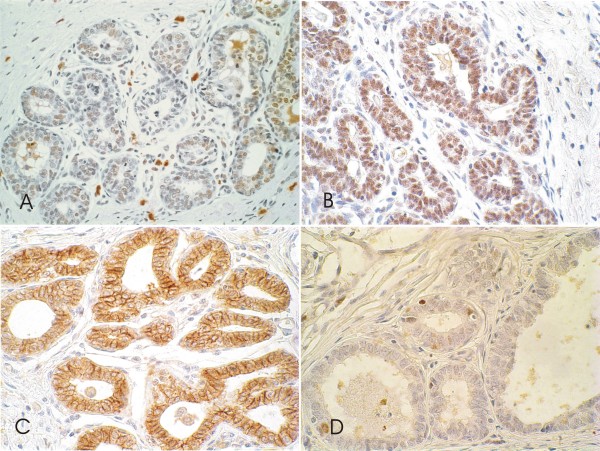
**Canine mammary biopsies with immunohistochemical staining of columnar cell lesion (CCL)**. Columnar cell change without atypia showing intense immunostaining for estrogen receptor (**4A**) and progesterone receptor **(4B)**. Flat epithelial atypia with moderate membrane E-cadherin reactivity **(4C) **and nuclear expression for Ki-67 **(4D), **600×.

CCC: columnar cell change; CCH: columnar cell hyperplasia without atypia; FEA: flat epithelial atypia; DH: ductal hyperplasia without atypia; ADH: atypical ductal hyperplasia;

The luminal epithelial cells with columnar change (five CCC and one CCH) showed a strong uniform nuclear immunopositivity for ER and PR antibodies in 50% and around 100% of cells respectively in all six cases studied (Figure 4A and 4B). Strong E-cadherin expression was detected in 5 out of 6 CCLs analyzed with a staining pattern similar to the adjacent normal TDLU. Within these specimens, three cases exhibited strong E-cadherin immunoreactivity in all cells and two exhibited strong immunoreactivity but with focal areas of reduced or absent immunostaining, typically affecting small isolated tufts of cells (Figure 4C).

The proliferation rate as measured by Ki-67 appeared higher in CCLs (mean 4.7%) than normal TDLU. Ki-67-positive cells were generally present but in low numbers in normal internal control tissue epithelium (<1% positive cells) (Figure 4D).

The CCLs were negative for cytokeratin 34βE-12, P53 and HER-2 in all six cases studied. Interestingly, P53 and HER-2 negative results were similar to those obtained with the associated benign and malignant tumors (two benign mixed tumors, one duct papilloma, one solid carcinoma and two ductal in situ carcinoma).

## Discussion

Columnar cell alterations of the canine mammary gland have been documented during microscopic analysis of breast tissues. A common component of most hyperplastic ductal lesions was columnar alteration (also referred to as columnar cell metaplasia) [12]. However, histopathological criteria that characterize columnar lesions in dogs and their malignant potential when detected as the sole histopathological abnormality following breast biopsy are undocumented.

The morphological appearance of the canine CCLs was very similar to the human breast. Hyperplastic breast lesions, such as ductal hyperplasias without and with atypia, have previously been described in the canine mammary gland [12]. To our knowledge, our study is the first to describe CCLs in the mammary glands of female dogs. In the present study we found CCLs in 53.1% of consecutive canine breast specimens. Lesions ranged from CCCs without atypia (61.1%) to FEA (38.8%).

Observational studies have revealed a relationship between CCLs and tubular carcinoma [15,16]. Columnar cell lesions, in particular those with nuclear atypia, may also co-exist with lobular in situ neoplasia. Flat epithelial atypia is associated with established DCIS more frequently than by chance [17,18].

Microcalcifications were also frequently found in association with canine CCLs, similar to human lesions. Although CCLs have long been recognized in the human breast under different names, their importance increased after more were diagnosed in mammographically detected lesions owing to microcalcifications [16,18]. Although mammography is not routinely used in canine species we supposed that such microcalcifications would have a radiological appearance similar to that in the human breast.

Myoepithelial/basal and some epithelial cells in human CCLs are positive for CK5/6 [19]. Though columnar cell lesions in human breast are usually negative for HER-2/neu, p53 and basal CK5/6 and CK14. Similar expression was found in all six cases studied, supporting the use of breast tumor biomarkers in this model. The immunohistochemical similarities between human and dog columnar cells also included, increased epithelial expression of the proliferation marker Ki-67/MIB1 and strong expression of hormonal receptors (ER and PR) and E-cadherin [13,20,21].

The canine species could be the most adequate model for new studies for cancer, due to the morphological and genotypic similarities of human lesions [20,22,23]. Spontaneous epithelial mammary lesions are common in dogs well before the age of onset of palpable mammary tumors [9,7]. Thus, spontaneous tumours of canine mammary glands have been proposed as comparative models for the study of human breast cancer, since these lesions share epidemiological, clinical, behavioural and antigenic features [23-25].

## Conclusions

Columnar cell lesions in canine mammary gland are pathologically and immunophenotypically similar to those in human breast. This may suggest that dogs are a suitable model for the comparative study of noninvasive breast lesions. Additional studies are needed to analyze the frequency of columnar cells lesions and their relationship to in situ carcinomas in canine mammary glands to provide a model for testing treatment modalities for mammary lesions and ultimately for clarifying patient management.

## Abbreviations

ADH: Atypical Ductal Hyperplasia; ALH: Atypical Lobular Hyperplasia; CCLs: Columnar Cell Lesions; CCC: Columnar Cell Change; CCH: Columnar Cell Hyperplasia; DCIS: Ductal Carcinoma In Situ; DH: Ductal Hyperplasia without atypia; ER: Estrogen Receptor; FEA: Flat Epithelial Atypia; LCIS: Lobular Carcinoma In Situ; PgR: Progesterone Receptor; TDLUs: Terminal Duct Lobular Units

## Competing interests

The authors declare that they have no competing interests.

## Authors' contributions

*EF *conceived the study, participated in the immunoassays, performed the statistical analysis and drafted the manuscript. *HG *participated in the study design and helped to draft the manuscript. *BS *carried out the immunoassays and participated in the design of the study. *GDC *participated in design and coordination of the study and helped to draft the manuscript. All authors read and approved the final manuscript.

## Pre-publication history

The pre-publication history for this paper can be accessed here:

http://www.biomedcentral.com/1471-2407/10/61/prepub

## References

[B1] Reis-Filho JS Lakhani SR The diagnosis and management of preinvasive breast disease: Genetic alterations in pre-invasive lesions Breast Cancer Res 2003 5 313 319 10.1186/bcr650 14580249 PMC314410

[B2] Abdel-Fatah TM Powe DG Hodi Z Lee AH Reis-Filho JS Ellis IO High frequency of coexistence of columnar cell lesions, lobular neoplasia, and low grade ductal carcinoma in situ with invasive tubular carcinoma and invasive lobular carcinoma Am J Surg Pathol 2007 31 3 417 426 10.1097/01.pas.0000213368.41251.b9 17325484

[B3] Fraser JL Raza S Chorny K Connolly JL Schnitt SJ Columnar alteration with prominent apical snouts and secretions: a spectrum of changes frequently present in breast biopsies performed for microcalcifications Am J Surg Pathol 1998 22 1521 1527 10.1097/00000478-199812000-00009 9850178

[B4] Schnitt SJ The diagnosis and management of pre-invasive breast disease: flat epithelial atypia - classification, pathologic features and clinical significance Breast Cancer Res 2003 5 263 268 10.1186/bcr625 12927037 PMC314429

[B5] Schnitt SJ Vincent-Salomon A Columnar cell lesions of the breast Adv Anat Pathol 2003 10 3 113 24 10.1097/00125480-200305000-00001 12717115

[B6] Dabbs DJ Carter G Fudge M Peng Y Swalsky P Finkelstein S Molecular alterations in columnar cell lesions of the breast Mod Pathol 2006 19 3 344 349 10.1038/modpathol.3800538 16400324

[B7] Vail DM MacEwen EG Spontaneously occurring tumors of companion animals as models for human cancer Cancer Invest 2000 18 781 792 10.3109/07357900009012210 11107448

[B8] Cassali GD Gobbi H Malm C Schmitt FC Evaluation of accuracy of fine needle aspiration cytology for diagnosis of canine mammary tumours: comparative features with human tumours Cytopathology 2007 18 3 191 196 17573766 10.1111/j.1365-2303.2007.00412.x

[B9] Antuofermo E Miller MA Pirino S Xie J Badve S Mohammed SI Spontaneous mammary intraepithelial lesions in dogs--a model of breast cancer Cancer Epidemiol Biomarkers Prev 2007 16 11 2247 2256 10.1158/1055-9965.EPI-06-0932 17982119

[B10] Martin de las Mulas J Ordás J Millán Y Fernández-Soria V Ramón y Cajal S Oncogene HER-2 in canine mammary gland carcinomas: an immunohistochemical and chromogenic in situ hybridization study Breast Cancer Res Treat 2003 80 3 363 367 10.1023/A:1024929730165 14503809

[B11] Smith GH Mammary cancer and epithelial stem cells: a problem or a solution? Breast Cancer Res 2002 4 2 47 50 10.1186/bcr420 11879561 PMC138717

[B12] Misdorp W Else RW Hellmén E Limpscomb TP Histological classification of the mammary tumors of the dog and the cat World Health Organization. International Histological Classification of Tumors of Domestic Animals 1999 2 2 Edited by OMS. Geneva: Switzerland

[B13] Simpson PT Gale T Reis-Filho JS Jones C Parry S Sloane JP Hanby A Pinder SE Lee AH Humphreys S Ellis IO Lakhani SR Columnar Cell Lesions of the Breast: The Missing Link in Breast Cancer Progression? A Morphological and Molecular Analysis Am J Surg Pathol 2005 29 734 746 10.1097/01.pas.0000157295.93914.3b 15897740

[B14] Wolff AC Hammond ME Schwartz JN Hagerty KL Allred DC Cote RJ Dowsett M Fitzgibbons PL Hanna WM Langer A McShane LM Paik S Pegram MD Perez EA Press MF Rhodes A Sturgeon C Taube SE Tubbs R Vance GH Vijver M van de Wheeler TM Hayes DF American Society of Clinical Oncology/College of American Pathologists Guideline Recommendations for Human Epidermal Growth Factor Receptor 2 Testing in Breast Cancer Arch Pathol Lab Med 2007 131 1 18 19548375 10.5858/2007-131-18-ASOCCO

[B15] Rosen PP Columnar cell hyperplasia is associated with lobular carcinoma in situ and tubular carcinoma Am J Surg Pathol 1999 23 1561 10.1097/00000478-199912000-00017 10584711

[B16] Pinder SE Reis-Filho JS Lesions Non Operative Breast Pathology: columnar cell J Clin Pathol 2007 60 12 1307 1312 10.1136/jcp.2006.040634 17182657 PMC2095568

[B17] Sahoo S Recant WM Triad of columnar cell alteration, lobular carcinoma in situ, and tubular carcinoma of the breast Breast J 2005 11 140 142 10.1111/j.1075-122X.2005.21616.x 15730461

[B18] Leibl S Regitnig P Moinfar F Flat epithelial atypia (DIN 1a, atypical columnar change): an underdiagnosed entity very frequently coexisting with lobular neoplasia Histopathology 2007 50 7 859 65 10.1111/j.1365-2559.2007.02700.x 17543075

[B19] Jensen KC Schaeffer DF Cheang M Montgomery K West RB Gilks CB Ross D Turashvili G Schnitt S Rijn M van de Characterization of a novel anti-fatty acid synthase (FASN) antiserum in breast tissue Mod Pathol 2008 21 12 1413 20 10.1038/modpathol.2008.163 18820672

[B20] Mac Laren BK Goobi H Schuyler PA Olson SJ Parl FF Dupont WD Page DL Immunohistochemical expression of estrogen receptor in enlarged lobular units with columnar alteration in benign breast biopsies: a nested case-control study Am J Surg Pathol 2005 29 1 105 8 10.1097/01.pas.0000146013.76881.d9 15613861

[B21] Turashvili G Hayes M Gilks B Watson P Aparicio S Are columnar cell lesions the earliest histologically detectable non-obligate precursor of breast cancer? Virchows Arch 2008 452 6 589 98 10.1007/s00428-008-0609-6 18437416

[B22] Genelhu MC Cardoso SV Gobbi H Cassali GD A comparative study between mixed-type tumours from human salivary and canine mammary glands BMC Cancer (Online) 2007 7 218 10.1186/1471-2407-7-218 PMC2233636 18045453

[B23] Lindblad-Toh K Wade CM Mikkelsen TS Karlsson EK Jaffe DB Kamal M Clamp M Chang JL Kulbokas EJ Zody MC Mauceli E Xie X Breen M Wayne RK Ostrander EA Ponting CP Galibert F Smith DR DeJong PJ Kirkness E Alvarez P Biagi T Brockman W Butler J Chin CW Cook A Cuff J Daly MJ DeCaprio D Gnerre S Grabherr M Kellis M Kleber M Bardeleben C Goodstadt L Heger A Hitte C Kim L Koepfli KP Parker HG Pollinger JP Searle SM Sutter NB Thomas R Webber C Baldwin J Abebe A Abouelleil A Aftuck L Ait-Zahra M Aldredge T Allen N An P Anderson S Antoine C Arachchi H Aslam A Ayotte L Bachantsang P Barry A Bayul T Benamara M Berlin A Bessette D Blitshteyn B Bloom T Blye J Boguslavskiy L Bonnet C Boukhgalter B Brown A Cahill P Calixte N Camarata J Cheshatsang Y Chu J Citroen M Collymore A Cooke P Dawoe T Daza R Decktor K DeGray S Dhargay N Dooley K Dooley K Dorje P Dorjee K Dorris L Duffey N Dupes A Egbiremolen O Elong R Falk J Farina A Faro S Ferguson D Ferreira P Fisher S FitzGerald M Foley K Foley C Franke A Friedrich D Gage D Garber M Gearin G Giannoukos G Goode T Goyette A Graham J Grandbois E Gyaltsen K Hafez N Hagopian D Hagos B Hall J Healy C Hegarty R Honan T Horn A Houde N Hughes L Hunnicutt L Husby M Jester B Jones C Kamat A Kanga B Kells C Khazanovich D Kieu AC Kisner P Kumar M Lance K Landers T Lara M Lee W Leger JP Lennon N Leuper L LeVine S Liu J Liu X Lokyitsang Y Lokyitsang T Lui A Macdonald J Major J Marabella R Maru K Matthews C McDonough S Mehta T Meldrim J Melnikov A Meneus L Mihalev A Mihova T Miller K Mittelman R Mlenga V Mulrain L Munson G Navidi A Naylor J Nguyen T Nguyen N Nguyen C Nguyen T Nicol R Norbu N Norbu C Novod N Nyima T Olandt P O'Neill B O'Neill K Osman S Oyono L Patti C Perrin D Phunkhang P Pierre F Priest M Rachupka A Raghuraman S Rameau R Ray V Raymond C Rege F Rise C Rogers J Rogov P Sahalie J Settipalli S Sharpe T Shea T Sheehan M Sherpa N Shi J Shih D Sloan J Smith C Sparrow T Stalker J Stange-Thomann N Stavropoulos S Stone C Stone S Sykes S Tchuinga P Tenzing P Tesfaye S Thoulutsang D Thoulutsang Y Topham K Topping I Tsamla T Vassiliev H Venkataraman V Vo A Wangchuk T Wangdi T Weiand M Wilkinson J Wilson A Yadav S Yang S Yang X Young G Yu Q Zainoun J Zembek L Zimmer A Lander ES Genome sequence, comparative analysis and haplotype structure of the domestic dog Nature 2005 438 7069 803 19 10.1038/nature04338 16341006

[B24] Strandberg JD Goodman DG Animal model of human disease: canine mammary neoplasia Am J Pathol 1974 75 1 225 8 4825616 PMC1910803

[B25] Warner MR Age incidence and site distribution of mammary dysplasias in young beagle bitches J Natl Cancer Inst 1976 57 57 61 1034018 10.1093/jnci/57.1.57

